# Natural Products Screening for the Identification of Selective Monoamine Oxidase-B Inhibitors

**DOI:** 10.9734/EJMP/2016/26453

**Published:** 2016-05-27

**Authors:** Najla O. Zarmouh, Samia S. Messeha, Faisel M. Elshami, Karam F. A. Soliman

**Affiliations:** 1 College of Pharmacy and Pharmaceutical Sciences, Florida A&M University, Tallahassee, Florida 32307, USA

**Keywords:** Parkinson disease, selective monoamine oxidase-B inhibitors, Psoralea corylifolia seeds, Phellodendron amurense, Glycyrrhiza uralensis, Ferula assafoetida

## Abstract

**Aims:**

Monoamine oxidase-B inhibitors (MAO-BIs) are used for the initial therapy of Parkinson’s disease. Also, MAO-BIs have shown to be effective neuroprotective agents in several neurodegenerative diseases. However, some concerns exist regarding the long-term use of these compounds. Meanwhile, natural compounds showed potential MAO-B selective inhibitions. To date, few selective natural MAO-BIs have been identified. Therefore, the current study is designed to identify plants with potent and specific MAO-B inhibition.

**Study Design:**

In this work, we utilized high throughput screening to evaluate the different plants ethanolic extract for their effectiveness to inhibit *recombinant human* (*h*)MAO-A and *h*MAO-B and to determine the relative selectivity of the top MAO-BI.

**Methodology:**

Recombinant human isozymes were verified by Western blotting, and the 155 plants were screened. A continuous fluorometric screening assay was performed followed by two separate *h*MAO-A and *h*MAO-B microtiter screenings and IC_50_ determinations for the top extracts.

**Results:**

In the screened plants, 9% of the extracts showed more than 1.5-fold relative inhibition of *h*MAO-B (RI_B_) and another 9% showed more than 1.5-fold relative inhibition of *h*MAO-A. The top extracts with the most potent RI_B_s were *Psoralea corylifolia* seeds, *Phellodendron amurense* bark, *Glycyrrhiza uralensis* roots, and *Ferula assafoetida* roots, with the highest RI_B_ of 5.9-fold. Furthermore, extensive maceration of the promising extracts led to increase inhibitory effects with a preserved RI_B_ as confirmed with luminescence assay. The top four extracts *h*MAO-BIs were equally potent (IC_50_= 1.3 to 3.8 μg/mL) with highly significant relative selectivities to inhibit *h*MAO-B (4.1- to 13.4-fold).

**Conclusion:**

The obtained results indicate that *Psoralea corylifolia* seeds, *Ferula assafoetida, Glycyrrhiza uralensis* roots, and *Phellodendron amurense* ethanolic extracts have selective inhibitions for human MAO-B. Investigating these plant extracts as natural resources for novel selective MAO-BIs may lead to the development of molecules that can be used in the therapeutic management of neurodegenerative diseases including Parkinson’s disease.

## 1. INTRODUCTION

In Parkinson’s disease and depression monoamine oxidase inhibitors (MAO-AIs and MOAIs) have been used in the therapy of these diseases. MAO-A and MAO-B are two isozymes that belong to the flavin-containing amine oxidases and can be found in astrocytes and the substantia nigra pars compacta (SNpc) neurons to metabolize monoamine neurotransmitters. In PD, MAO-BIs are used to increase neurotransmitter dopamine (DA), reduce oxidative stress level and relieve the psychomotor disease symptoms [1]. DA is known to be metabolized by both isozymes [2], MAO-B is more specific in metabolizing the already depleted DA in the SNpc of the PD patients [3]. Additionally, the activity of MAO-B is elevated up to three-fold in PD and Alzheimer’s disease (AD), compared to normal levels [4]. That MAO-B elevation [1,5] with the co-localized of active MAO-A isozyme can potentially aggravate oxidative stress in the aging patients. Both MAOs activities produce abnormally high amounts of hydrogen peroxide (H_2_O_2_) and aldehydes that are neurotoxic. Those by-products potentially damage proteins, nucleic acids, lipids and activate apoptotic pathways [6]. Unfortunately, the aldehyde dehydrogenase enzyme that metabolizes the neurotoxic aldehydes produced by the active MAOs was found to be genetically deficient in PD patients’ SNpc [7,8]. Other oxidative stress defense enzymes may also become limited with the overwhelming reactive species produced. Consequently, these toxic byproducts, particularly of active MAO-B, can potentially accumulate in neurons and astrocytes leading to cell death and aggravating neurodegeneration.

While MAO-AIs are usually associated with concerns about food and drug interactions that lead to rare but serious side effects (the cheese effect and serotonin syndrome) [9,10], MAO-BIs were found ideal for the management of PD as in the case of selegiline (Deprenyl^®^) (DEP). These inhibitors were proven to be clinically efficient for decades as they delayed the need for L-dopa in PD management. Selective MAO-BIs may also inhibit the conversion of nontoxic xenobiotic substrates to neurotoxins in the brain, such as the MPTP conversion to its neurotoxic product MPP+. Also, MAO-BIs exert anti-apoptotic and other multifunctional neuroprotective activities [11] that may lead to extension of the PD patients’ life expectancy [8]. Moreover, MAO-BIs such as deprenyl were reported beneficial in other neurological disorders such as cerebrovascular ischemia, Tourette syndrome, narcolepsy, and AD [12].

Although this may sound ideal for the MAO-BI deprenyl, in neurological diseases, some concerns exist with the long-term use of this medication. Recent evidence of neurotoxic metabolite of deprenyl, L-methamphetamine, showed contradictions in their antiparkinsonian action *in vitro* [13], and some attributed rare cases of tolerance or dependence development on some MAOIs to their amphetamine-like metabolites structures [14]. Meanwhile, the currently available MAO-BIs are synthetic compounds (such as deprenyl and rasagiline) that share common structure of the propargyl functional group which is responsible for MAO inhibition and neuroprotection [2]. On the other hand, it was reported that potent and selective MAO-BIs in nature are commonly found to include flavonoids, β-carbolines, xanthines, and alkaloids [15]. Therefore, new natural structures may promote the discovery of new lead compounds with unique properties as in the classical MAO-BIs. To recognize if the total phytochemical constituents of plant extracts also have the ability to selectively inhibit human MAO-B, high-throughput screening (HTS) was conducted on both isozymes. Identifying the plants with the most selective MAO-B inhibitory properties may further reveal unique phytochemical structure properties with multifunctional neuroprotective and neurorescue properties, beneficial to neurodegenerative diseases such as PD.

## 2. METHODOLOGY

### 2.1 Materials

The *h*MAO-A and *h*MAO-B isozymes, produced in BTI-TN-5B1-4 insect cells containing human cDNA, and their analyzed active units (U), were purchased from Sigma-Aldrich (St. Louis, MO, USA). Isozymes stocks were diluted with 1% of 1 M HEPES in Hank’s Balanced Salt Solution (HBSS) (pH 7.4) and aliquots stored at −80°C for single use. Standards of pirlindole, a reversible inhibitor for MAO-A (RIMA), deprenyl (DEP), an irreversible MAO-BI, and cell culture media and supplements were also purchased from Sigma-Aldrich. Different plant dry parts (leaves, stems, roots, petals, barks, resins, or whole herbs) were purchased from and identified by their trades companies including, East Earth Trade Winds (Redding, CA, USA), Mountain Rose, Herbs (Eugene, OR, USA), Mayway Corp. (Oakland, CA, USA), Monterey Bay Spice Comp. (Watsonville, CA, USA). The plants used were not specific to one region. Western blotting equipment and reagents were purchased from Bio-Rad Laboratories (Hercules, CA, USA) and BCA Protein Assay Kit from Peirce (Rockford, IL, USA). Amplex™ Red MAO Assay Kit was purchased from Molecular Probes by Life technologies™ (Eugene, OR, USA), and tyramine HCl from Santa Cruz Biotechnology (Dallas, TX, U.S.A.). MAO-Glo™ Kit was purchased from Promega Inc. (Madison, WI, USA).

### 2.2 Ethanolic Extraction

Plants used in this study were extracted for screening for their *h*MAO-A and *h*MAO-B inhibiting potentials, and the top active extracts (potent and selective at 1 mg/mL) were further extensively extracted. Briefly, 155 different dry plant parts were used (e. g. leaf, stem, root, petal, bark, resin, and herb). Each defined amount of 250 mg was ground to fine powders, homogenized in 99.95% ethanol and macerated once for 50 mg/mL extracts. The top four active plants, of 8 g each, were subject to repeated maceration with mild agitation as the used ethanol solvent was exchanged every 24 h and evaporated in a fume hood for ten days to get the crude extract. Only *Ferula assafoetida* resin (FAR) was subject to 80°C evaporation for a short time using a rotary evaporator for speed dry. All labeled ethanolic extracts (EEs) were stored in airtight glass containers at −20°C in the dark until use.

### 2.3 Proteins Verification and Method Validation

#### 2.3.1 Western blotting

Western blotting was used to verify MAO isozymes. Human dopaminergic neuroblastoma cell line of SH-SY5Y was used as a positive control containing isozymes, MAO-A [16], and MAO-B, as in the anti-MAO-B datasheet. The cells were obtained from American Type Culture Collection (CRL-2266) (Manassas, VA, USA) and were cultured in DMEM with 10% fetal bovine serum, 100 IU per mL penicillin/streptomycin. To lysate the cells, we used RIPA buffer/protease inhibitor (4°C) with freezing and thawing cycles.

To assure equally loaded amounts in micrograms, we performed the BCA protein assay, and the Bio-Tek Synergy HTX Multi-Reader set to 562 nm for analysis. All samples were prepared with 2 × Laemmli sample buffer-2.5% mercaptoethanol loading buffer for 12 μg per lane. Proteins were denatured using heating block for 3–5 min at 100°C before loading and separated using 1D SDS-PAGE gel electrophoresis of 10% Tris-HCl gradient at 200 V for 55 min. Gels were wetly transferred to nitrocellulose membranes at 100 V for 75 min. Primary antibodies used were rabbit monoclonal anti-MAO-B antibody [EPR7103] (Abcam; ab125010), rabbit monoclonal anti-MAO-A antibody [EPR7101] (Abcam; ab126751) with 1–2:1000 ratio each in cold skim milk. Rabbit anti-β-actin antibody (Abcam; 8227) was used for control. Secondary antibodies were goat anti-rabbit IgG H&L HRP-conjugated probes (Abcam; ab6721). The signal was detected using Supersignal^®^ West Pico Chemiluminescent Substrate from Thermo Scientific, Peirce Biotechnology (Rockford, IL, USA) and VersaDoc imaging system using CCD camera (Bio-Rad; Hercules, CA, USA).

#### 2.3.2 Substrate metabolism with time

In this experiment, substrate concentrations and time required for maximum detectable *h*MAO-A and MAO-B activities were validated; optimal parameters were determined using the continuous Amplex Red fluorometric assay. In brief, *h*MAO-A and B (0.7 U/mL; 0.07 U per reaction) activities were assayed using p-tyramine HCl (tyr.HCl) and benzylamine HCl (benz. HCl) as substrates, respectively. Different substrate volumes of 25 μL of 4 × the final concentrations were added into black opaque 96-well microplates for their related isozyme assay. Added substrate final concentrations ranged from 0 to 0.8 mM with *h*MAO-A, and from 0 to 3 mM with *h*MAO-B, as buffers substituted substrates in control wells. In the dark, the fluorometric reagent was prepared as 4 × the final concentration of 200 μM Amplex Red 1 U/mL and horseradish peroxidase (HRP type-II) in PBS (pH 7.4). Freshly prepared reagent of 25 μL was added to each well and the reaction was initiated by adding 50 μL of 2 × isozyme final concentration to the different related substrate concentrations and controls in the wells. Immediately, the fluorescent signal (AFU) of the reactions kinetics with time was read at various time intervals (minutes then hours) at RT. Pre-plate for time zero and post-plate readings for different time intervals were obtained by subtracting the time zero pre-plate reading to monitor the increase as an indicator for the product resorufin continuous accumulation. The AFU excitation resorufin was at 530 nm, and its read fluorescence detection was at 590 nm using Synergy HTX Multi-Reader (Bio-Tek).

#### 2.3.3 H_2_O_2_ scavenging activity, autoxidation, and resorufin quenching

Determining maximum H_2_O_2_ produced within 1 h of incubation at RT was accomplished by interpolating maximum AFU from the H_2_O_2_ linear standard curve of ranged 0–5 μM (R^2^ of 99.3%) using GraphPad Prism software. Values of the blank wells without H_2_O_2_ or enzymes were subtracted from all their corresponding test values. MAO total H_2_O_2_ production was at a maximum of 0.9±0.01 nmol (4.5±0.07 μM). Thus, the scavenging activities were tested for a maximum of 5 μM at RT. Freshly prepared H_2_O_2_ was added as 4 × the final concentration to 2 × the final extract concentrations equivalent to MAOs assays. The quenching ability of the Amplex Red product resorufin by the extracts was tested. Based on preliminary studies, resorufin was added as 4 × the final concentration of 20 μM to 1.3 × the final extract concentrations equivalent to MAOs assays. In autoxidation, the reactions were measured with the same method as scavenging activities except substituting H_2_O_2_ with used reaction buffer and calculated separately as folds of signal increase. Any extract with ≥ 50% scavenging or ≥ 30% quenching activities were excluded from the *h*MAO-A and *h*MAO-B inhibition extract screenings.

### 2.4 *h*MAO-A and *h*MAO-B Fluorometric Microtiter Screening

MAOs activities were assayed using an extremely sensitive continuous fluorometric assay containing Amplex Red (10-acetyl-3, 7-dihydroxyphenoxazine) reagent. The enzymatic H_2_O_2_ was measured with and without extracts or standards. In addition to random plant selection, some plants were chosen based on our previous work on *h*MAO-B natural inhibitors [17]. Briefly, each of the 155 EEs was diluted in PBS (pH 7.4) in black 96-well microplate to equally make 4 × the final concentration of 1 mg/mL (n= 2). *h*MAO isozymes on ice with 4 × the final concentration, 0.7 U/mL each, were used. The *h*MAO-A and *h*MAO-B (25 μL) were separately added to 25 μL EEs or buffer for control and incubated 30–40 min at RT. For the top four extracts IC_50_s determination, 8 × working solutions in PBS (pH 7.4) were serially diluted for at least ten points before adding the enzymes as mentioned earlier. Control groups were tested with and without maximum ethanol of 1.25%. Buffer solution substituted the enzymes in the correspondent blank wells.

The 4 × working solution of Amplex Red reagent was freshly prepared as mentioned earlier in the substrate metabolism optimization method. The previously optimized 4 × final concentration of 0.5 mM tyr HCl (for *h*MAO-A) and 3 mM be HCl (for *h*MAO-B) were prepared. Each substrate was mixed with Amplex Red reagent at 1:1 ratio. A 50 μL of each mixed solution was added to its corresponding enzyme/extract wells to make the required final extract concentrations. Fluorescent resorufin product was quantified at different time intervals as plates were read at an excitation/emission of 530/590 nm using Synergy HTX Multi-Reader (Bio-Tek, USA). Time zero pre-plate and post-plate readings, at times of 60 min each, were obtained. Percent enzyme inhibition and relative inhibition (RI_B_) were determined for all extracts. In comparison to the related control, any extract that inhibited *h*MAO-B to less than 85% or showed > 1.5-fold ratio RI_B_ was pointed out. The same was done to the top relative inhibitors against *h*MAO-A (RI_A_). Only extracts that ranked the most potent against *h*MAO-B were further evaluated for IC_50_s as with DEP and pirlindole standard controls.

### 2.5 Confirmation by a Luminescence Assay

A luminescence assay, using the MAO-Glo™ Kit, was used with DEP standard to ensure preserved RI_B_. Briefly, 12.5 μL of 4 × the final concentrations of 20 μg/mL of each extensively extracted plant (PCSEE, PABEE, FAREE, and GUREE) or 5 μg/mL DEP were added to white opaque 96-well microplate. Fresh 25μL of 2 × the final concentration of 0.9 U/mL *h*MAO-A and *h*MAO-B isozymes in reaction buffer (pH 7.4) were incubated with the extracts for 30 min at RT. Controls used were with and without ethanol (0.1%). Reaction buffer substituted each corresponding isozyme to make the blank wells. Based on Valley’s method [18] and our preliminary optimizations, 12.5 μL of 4 × the final concentration of 40 and 4 μM of luciferin derivative substrate for *h*MAO-A and *h*MAO-B reactions, were added respectively. The reaction was incubated for 60 min at RT. Reporter luciferase detects reagent of 50 μL per well was added. After 30 min of incubation, produced arbitrary light units (ALU) were detected using Synergy HTX Multi-Reader (Bio-Tek).

### 2.6 Statistical Analysis

Analysis performed by GraphPad Prism Software v6.02 (San Diego, CA, USA). Data points were presented as the mean ± SEM. IC_50_s values were interpolated from normalized data by the asymmetric sigmoidal curve and averaged from at least two experiments. One-way and two-way ANOVA were performed followed by multiple comparisons tests (Dunnett’s after one-way ANOVA, and Sidak’s after two-way ANOVA) to determine the significance of the difference between each two or more groups as expressed in the figures. In this investigation, relative inhibition (RI_B_) which is the ratio of %*h*MAO-A activity/%*h*MAO-B activity at a particular concentration and relative selectivity (RS_B_) which is the ratio of *h*MAO-A IC_50_/*h*MAO-B IC_50_ were measured.

## 3. RESULTS

### 3.1 *h*MAO-A and *h*MAO-B Verification

**Fig. 1:**
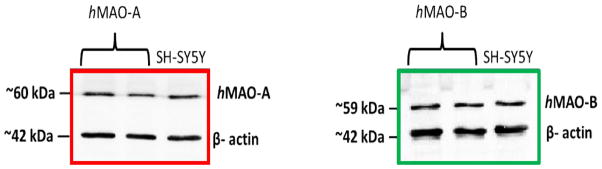
Verification of the used isozymes *h*MAO-A and *h*MAO-B identities by Western blotting using equally loaded proteins and rabbit monoclonal antibodies (anti-MAO-A, anti-MAO-B and anti-β-actin). SH-SY5Y cells (12 μg) were used as positive control for both isozymes. Bands were detected by HRP-conjugated anti-rabbit secondary antibody

Both *h*MAO-A and *h*MAO-B identities were verified using Western blotting. The human MAOs antibodies and β-actin Western blotting successfully identified both *h*MAO-A and *h*MAO-B sample proteins at about ~60, ~59, and ~42 KDa, respectively (Fig. 1). High intensity detected bands for *h*MAO-A and *h*MAO-B matched the human neuroblastoma SH-SY5Y cells positive controls at their molecular weights.

### 3.2 *h*MAO-A and *h*MAO-B Assay Method Validation

**Fig. 2:**
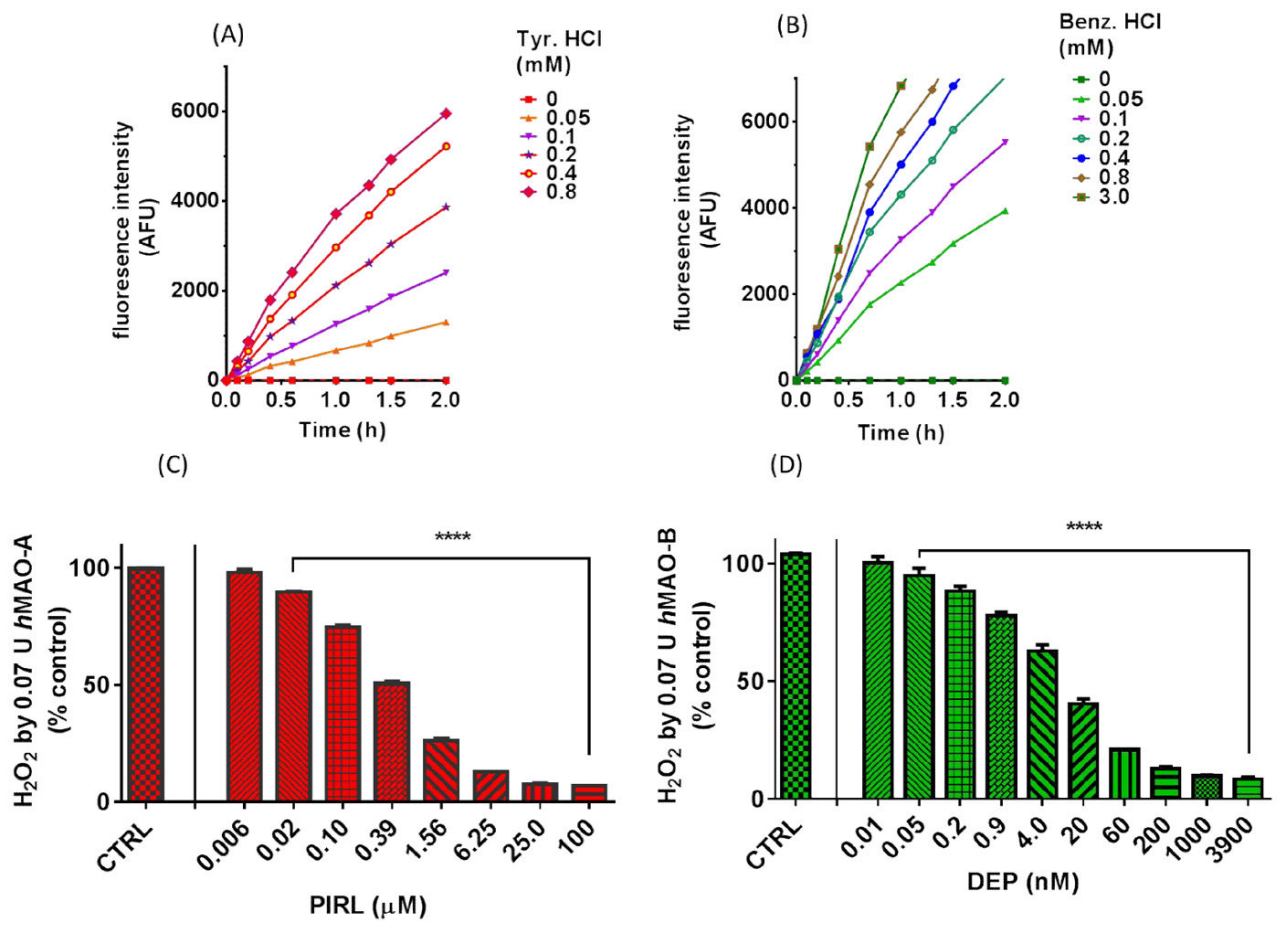
Fluorometric assay validation for screening; time and substrate concentration optimization with (A) *h*MAO-A and (B) *h*MAO-B isozymes at RT. AFU: Arbitrary Fluorescence Units. Progression curves with best maximum linearity in the presence of different substrate concentrations. Optimized conditions of isozymes were inhibited dose-dependently by selective standard inhibitors: (C) MAO-AI pirlindole (PIRL) and (D) MAO-BI deprenyl (DEP) Statistical analysis was presented as the mean ± SEM, n= 3. The significance of difference between a standard and its control reaction was determined using one-way ANOVA followed by Dunnett’s multiple comparisons test. **** p < 0.0001

To optimize the required time of incubation and substrates concentrations for the used isozymes amounts, an enzyme-progression curve with different substrate concentrations was performed before the screening. A proportional increase of AFUs was detected by the used 0.07 U isozyme (0.7 U/mL) at its initial linear rate of reaction (Fig. 2) at RT. AFU, as an H_2_O_2_ indicator, increased linearly (R^2^= 99.33%) with a maximum of 6304±25 AFU with time and substrates concentrations within 2 h by *h*MAO-A (Fig. 2A), and 1 h by *h*MAO-B (Fig. 2B). For optimum isozymes activities, tyr. HCl concentrations of 0.5 to 0.8 mM (Fig. 2A), and benz. HCl up to 3 mM were required (Fig. 2B). Using the optimized conditions with standard selective inhibitors of MAO-AI pirlindole and MAO-BI DEP (Fig. 2C and D); DEP and pirlindole selectively and dose-dependently inhibited their isozymes (DEP *h*MAO-A IC_50_= 1.2±0.5 μM and *h*MAO-B IC_50_= 10±10 nM, and pirlindole *h*MAO-A IC_50_= 0.24±0.05 μM and *h*MAO-B IC_50_= 262.2±5.8 μM). To exclude other possible interactions that may interfere with the *h*MAOs assays, H_2_O_2_ scavenging, autoxidation, and quenching activities were pre-tested. Scavenging and quenching activities tests were performed on all 155 prepared extracts. That led to excluding 30 extracts from the *h*MAO-A and *h*MAO-B inhibition screen to eliminate their interferences.

### 3.3 Microtiter Screening for *h*MAO-B Relative Inhibition (RI)

**Fig. 3:**
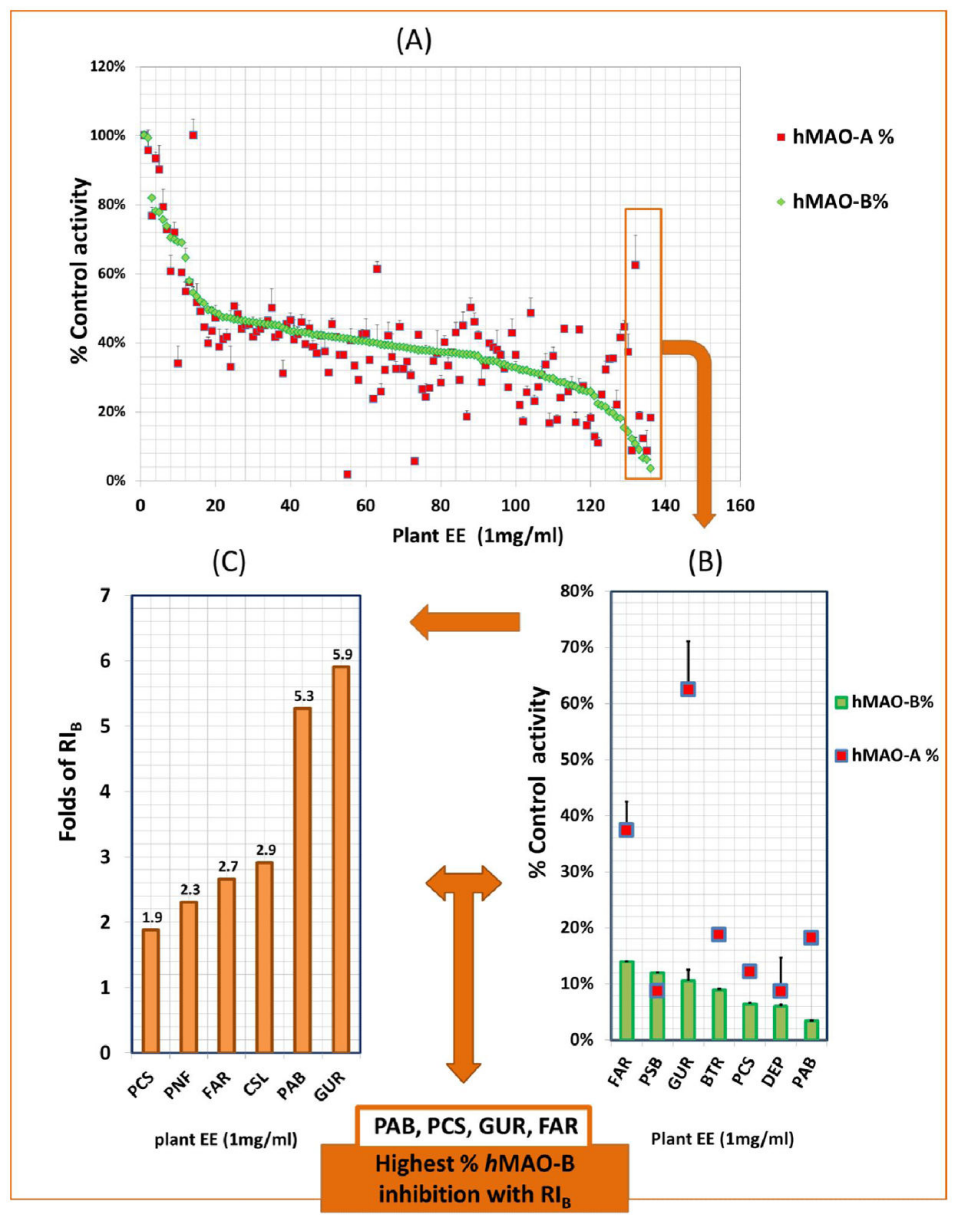
Plant high throughput screening to determine the top relative inhibitors of recombinant human monoamine oxidase-B (*h*MAO-B) (RI_B_): (A) 132 ethanolic plant extracts of 1 mg/mL were tested for both *h*MAO-A and *h*MAO-B inhibitory effects (B) The top effective six extracts inhibited > 85% of *h*MAO-B activity. (C) The top six extracts with the highest RI_B_ of >1.8-fold The most effective inhibitors with the highest RI_B_ in this screen were Glycyrrhiza uralensis (GUR); Psoralea corylifolia seeds (PCS) Phellodendron amurense barks (PAB), and Ferula assafoetida resin (FAR). Data points compared to standard deprenyl (DEP) were expressed as mean ± SEM, with n= 2. RI_B_= %hMAO-A/%hMAO-B

To determine the potential of the different plant extracts to exhibit potent RI_B_, two separate *h*MAO-A, and *h*MAO-B inhibition microtiter screenings were conducted using the continuous fluorometric assay (Fig. 3) as previously recommended for HTS [19]. After excluding extracts with the H_2_O_2_ scavenging or quenching activities, 132 out of 155 EEs total were tested for both isozymes and ranked as *h*MAO-BIs from low to high (Fig. 3A). The figure shows the different inhibition efficacies and relative inhibitions; extracts were effective against *h*MAO-B (green dots curved down), *h*MAO-A (red dots scattered away lower than the green curve), both, or no inhibitions. Interestingly, the screening elucidated 9% of the 132 plants extract with >1.5-fold *h*MAO-B relative inhibition (RI_B_) (Table 1), and other 9% of the extracts exerted >1.5-fold *h*MAO-A relative inhibition (RI_A_) (Table 2). The screen results indicated that plants have the potential to have a collectively selective *h*MAO-A and *h*MAO-B inhibiting activities (at least p = 0.05). These particular plants may contain more selective *h*MAO-B or *h*MAO-A inhibitors than the ones without different significant inhibitions.

The first step in our *h*MAO-B inhibition selectivity screen was to determine the percent inhibitory effects against the *h*MAO-B activity. The most effective inhibitors of >85% *h*MAO-B activity in Fig. 3B ranks were *Phellodendron amurense* barks (PAB) > *Psoralea corylifolia* seeds (PCS) > *Baptisia tinctoria* roots (BTR) > *Glycyrrhiza uralensis* roots (GUR) > *Paeonia suffructicose* roots and barks (PSB) > *Ferula assafoetida* resins (FAR). Further in the determination of RI_B_, the six top ranged RI_B_ extracts were partially different (Fig. 3C). Although PAB showed the most potent *h*MAO-B inhibition, the extract with the highest RI_B_ was GUR (5.9-fold). That was followed by PAB, *Camellia sinensis* leaves (CSL), FAR, *Piper nigrum* fruits (PNF), and PCS. From Figs. 3A & B, the screened extracts with shared characters of activities against *h*MAO-B and RI_B_ (PAB, PCS, GUR, and FAR) were selected for further selectivity determination. That method of selection based on the top six-ranked screen plants inhibitory efficacy and RI_B_ is to include selective *h*MAO-B inhibition properties that are hidden by extract high inhibitory effects.

### 3.4 Confirmation of Relative *h*MAO-B Inhibition (RI)

**Fig. 4:**
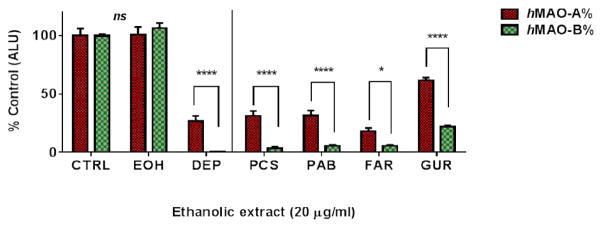
Luminescence assay confirmation of relative inhibition of *h*MAO-B (RI_B_) by extensively extracted plants of *Psoralea corylifolia* seeds (PCS), *Phellodendron amurense* (PAB), *Ferula assafoetida* resin (FAR) and *Glycyrrhiza uralensis* (GUR). ALU: arbitrary light units. Controls activities were compared with ethanol (EOH) and standard MAO-B inhibitor selegiline (DEP) at 5 μg/mL. All four extracts effectively inhibited *hMAO*-B more than *h*MAO-A Data points were presented as the mean ± SEM, with at least n= 3. The significance of difference between the two isozymes was determined using two-way ANOVA followed by Sidak’s multiple comparisons test. * p ≤ 0.05, **** p < 0.0001

To confirm the screening results and the preservation of RI_B_ of the selected top four extracts (GUR, PAB, PCS, and FAR) after extensive maceration, we used a non-H_2_O_2_-dependent luminescence assay (Fig. 4). The used ethanol had no effects on the assay. All tested extensively extracted EEs of only 20 μg/mL exerted an equally effective *h*MAO-B inhibition (p > 0.05) by > 70% of the 0.4 U isozymes activities. Moreover, the extracts showed very significant high RI_B_ activities (8.5-, 5.6-, 3.3-, 2.8-fold for PCS, PAB, FAR, and GUR, respectively (p ≤ 0.05 and 0.0001). The results indicate that the screen was successful in finding effective RI_B_s. Also, extracts efficacies of inhibition are relatively high, and their selectivities to inhibit *h*MAO-B had not been altered nor masked by the extensive extraction. Notably, extensive extraction showed an alteration in rankings of extracts with PCS higher inhibition against *h*MAO-B (p < 0.01) than GUR.

### 3.5 *h*MAO-B Relative Selectivity (RS_B_)

**Fig. 5:**
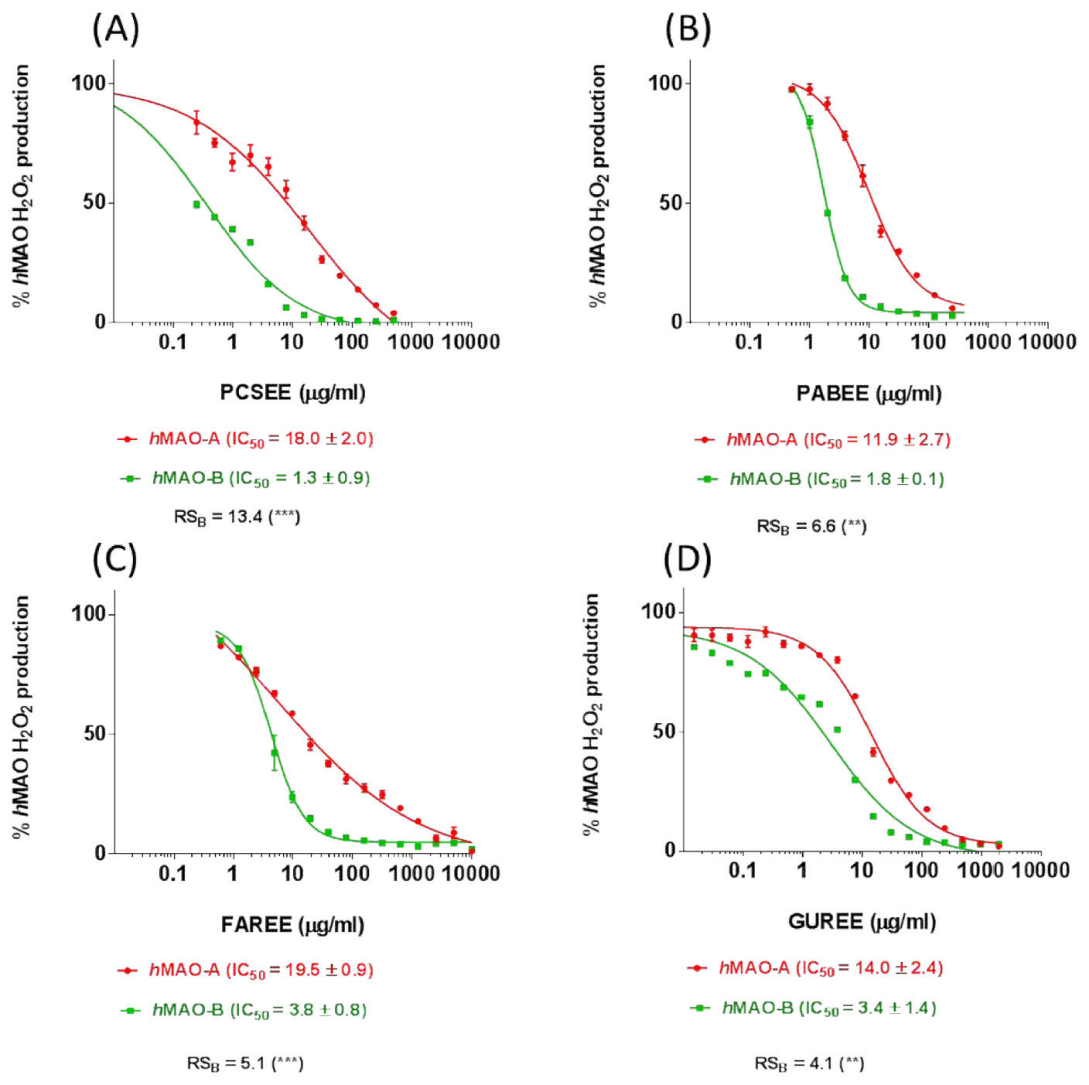
*h*MAO-A and *h*MAO-B inhibitory potencies and *h*MAO-B relative selectivity (RS_B_) of the extensively macerated ethanolic extract of (A) *Psoralea corylifolia* (PCSEE), (B) *Phellodendron amurense* barks (PABEE), (C) *Ferula assafoetida* (FAREE), and (D) *Glycyrrhiza uralensis* (GUREE). All extracts were likewise potent MAOs inhibitors with a significantly high RS_B_ The percent points were presented as the mean ± SEM, n= 4. IC_50_ ± SEM values were averaged from two experiments. Significance of difference between the two isozymes IC_50_s for each extract was determined using two-way ANOVA followed by Sidak’s multiple comparisons test. ** p < 0.01,*** p < 0.001

To determine the most selective extract among the four extensively macerated plants with ethanol, the relative selectivity (RS_B_) of each of GUREE, PCSEE, PABEE, and FAREE was investigated using Amplex Red assay of both isozymes (Fig. 5). No significant difference was observed between controls with and without the used ethanol concentrations. With a similar X-axes scale, all tested EEs showed a concentration-dependent *h*MAO-A and *h*MAO-B inhibitory potencies with clear RS_B_s. The extracts showed no significant different *h*MAO-B inhibitory potencies from each other (P > 0.05). Nonetheless, the RS_B_ of each of the four extracts was highly significantly different (p < 0.01 and 0.001). Specifically, the most selective *h*MAO-B inhibitors among the four-tested EE were PCSEE and FAREE, with more significant difference RS_B_s (p < 0.001) than GUREE and PABEE (p < 0.01). The results obtained also indicate preserved RS_B_s with increased potencies against *h*MAO-B with the extensive maceration.

## 4. DISCUSSION

Plant extracts ability to inhibit human MAO-B selectively was investigated by microtiter screening of 132 ethanolic plant extracts out of the 155 extracts. The initial screen indicated the high potential of plant extracts that contain varieties of selective *h*MAO-BIs, *h*MAO-AIs, and non-selective *h*MAOIs. The screen designated the abundance of selective MAO-A and MAO-B inhibitors in nature. While it is less relevant for PD, and thus beyond the scope of this work to investigate *h*MAO-A inhibitors, our focus was on the plants that specifically inhibit *h*MAO-B. *Psoralea corylifolia* seeds, *Phellodendron amurense* barks, *Glycyrrhiza uralensis* roots*, and Ferula assafoetida* resin ethanolic extracts stood out as potent and selective *h*MAO-B inhibitors. Regardless of their extensive extraction and the used assay, the four extracts consistently showed higher relative inhibition of *h*MAO-B than *h*MAO-A, which indicates an intrinsic selectivity to inhibit *h*MAO-B. On the other hand, the further extensive extraction dramatically enhanced the extracts potencies. Particularly at high concentrations and similar to the used standards of DEP and pirlindole, the high extracts potencies concealed, but did not alter, their preserved *h*MAO-B relative inhibition (Figs. 4 and 5).

The obtained four plant ethanolic extracts preliminary RI_B_s and conclusive RS_B_s were not due to their effects on H_2_O_2_ as confirmed with the luminescence assay. H_2_O_2_ scavenging activities or redox properties would equivalently reduce the total H_2_O_2_ in both assayed isozymes at the same extract concentration. Also, the H_2_O_2_ scavenging activity can alter the inhibition selectivity from *h*MAO-B to *h*MAO-A, which produces less H_2_O_2_ at 1 h reaction. The Amplex Red assay used for this screen is a highly sensitive one-step reaction method with a stable detection reagent product. It was previously evaluated for HTS and proposed over other conventional HPLC method for its convenience and continuity [19]. The use of the endogenous substrate tyramine and measuring the cytotoxic enzymes product H_2_O_2_ is advantageous as it mimics the biological reactions within the body. In contrast with the luminescence assay, *h*MAO-B very high luciferin derivative substrate affinity (4 μM) may not represent natural neurotransmitters affinities as benz. HCl does in the fluorometric assay. However, our used fluorescence assay led to eliminating many extracts from the screening as it was not suitable to detect MAOIs in extracts with H_2_O_2_ scavenging activities because of the extract direct interferences with the enzymatic H_2_O_2_.

A previous report from our laboratory showed that these four plants have *h*MAO-B inhibition, which supports the current findings [17]. In this report, extracts were only tested against one isozyme, the *h*MAO-B. The current study reveals that the four plants studied have high selectivity to the *h*MAO-B inhibition, which distinguishes them from plants with non-selective or *h*MAO-A selective inhibitions. Research on the selectivity of the studied plants against either MAOs isozymes has not been reported before. We believe that these findings of the ranked plants are of significant importance when searching for natural resources of selective MAO isozyme inhibitors. The chance to find phytochemicals with selective MAO-B inhibition is believed to be higher in extracts with selective MAO-B inhibition. Our findings of the MAO-B inhibitory selectivities were validated using two methods. Therefore, the consistency of the obtained results supports that there are extracts that are selective MAO-BIs.

In this work, the investigated *Ferula assafoetida* resin (*aka* stinking assa; family Apiaceae) showed high potency and selective inhibition of *h*MAO-B. This resin has been used as a spice and a phytomedicine around the globe for centuries. In the folklore medicine, it is mostly used in asthma, gastrointestinal disorders, and neuronal disorders [20]. In recent reports, the resin improved memory and learning in rats [21], and exhibited neuroprotection and nerve stimulation in mice peripheral neuropathy [22], and anticonvulsant properties [23]. FAR contains bioactive phytochemicals such as polysulfides, sesquiterpenes, sesquiterpene-coumarins, diterpenes, phenolics, and flavonoids [20,24]. Its coumarin umbelliprenin showed anti-inflammatory properties [20], while ferulic acid showed anti-atherosclerotic, antioxidant, and neuroprotective properties [25] and became a candidate for AD [26]. Therefore, investigations on the resin concerning PD need to be considered.

In addition, the seeds of *Psoralea corylifolia* (*aka*, Bu Gu Zhi or Babchi; family Leguminosae) are important in traditional Chinese and Ayurvedic medicines [27]. PCSEE was one of the most potent and selective *h*MAO-BI using our fluorometric screening assay. Our PCS findings are supported by our previous investigations on its *h*MAO-B inhibitory potency tested spectrophotometrically [17], and its selectivity for *h*MAO-B using a luminescence assay [28]. Previous PCS screened extracts for active constituents revealed that the ethanolic extract composes more medically active compounds than some other PCS extracts, which makes it a better candidate for novel phytomedicines [29]. PSCEE is rich in benzopyrone structure constituents including coumarins and flavonoids. PCS furocoumarins psoralen and isopsoralen showed *rat* MAOs activities inhibitions [30], which were supported by total furocoumarins potent antidepressant effects on mice [31]. PCS also contains isoflavones, which have been used as dietary supplements in various diseases, including osteoporosis, cognitive dysfunction, cardiovascular disease, and inflammation [32], which are close to PCS multifaceted properties [33–35]. We previously investigated bavachinin and genistein flavonoids constituents of PCS. Bavachinin exhibited a selective *h*MAO-B inhibition [28] while isoflavone genistein was similarly potent but less selective against *h*MAO-B [36]. Moreover, PCSEE contains monoterpenes that protected against the MAO-B substrate 1-methyl-4-phenyl-1,2,3,6-tetrahydropyridine (MPTP) SN cell damage and MPTP-induced motor deficits in PD model [37], inhibited DA and norepinephrine (NE) transporters [38], and showed antidepressant effects with catecholamine neurotransmitters regulation [39,40]. The PCS extracts were also neuroprotective against the MPTP precursor MPP+ [38] and the nitropropionic acid (3-NP) induced cytotoxicity and mitochondrial dysfunction [41]. Although the seeds are used in dermatological disorders health supplements [33] and increasingly investigated on *in vitro* and animal models, the extract and its phytochemicals clinical effects on degenerative diseases are yet to be clinically considered. From our results, the observed association between PCS constituents MAO-B inhibitions and the extracts neuroprotection in the previous reports suggests more investigations for potential beneficial PCS phytochemicals for PD.

Also*, Phellodendron amurense* (*aka* Amur cork tree; family Rutaceae) is a meagerly investigated Chinese medicinal plant. In our study, its bark ethanolic extract clearly was a selective *h*MAO-BI as its potent inhibition was previously spectrophotometrically confirmed [17]. The plant constituted alkaloids such as phellodendrine, palmatine, jatrorrhizine, and berberine [42,43] where the later displayed safe antidepressant-like activities in mice by the possible mechanism MAO-A inhibition and increasing DA, NE and serotonin brain levels [44,45]. PAB is high in the flavone tetramethyl-o-scutellarin, and the triterpenoids limonoids [42]. Limonoid obacunone was found neuroprotective in glutamate-induced neurotoxicity *in vitro* [46]. In clinical studies, PAB extract supplement safely reduced cortisol [47], and relieved mild anxiety in women [48]. Also, PAB inhibited pro-inflammatory cytokines [49,50] and protected from prostate tumors progression [51], property found in some MAO-AIs [52]. Based on our results and literature, there is a lack of knowledge on MAO-B inhibition and selectivity benefits of PAB extracts and phytochemicals. Further studies on PABEE as MAO-BI source for PD are highly recommended.

The roots of *Glycyrrhiza uralensis* (aka Chinese licorice; family Leguminosae) is another commonly used medicinal plant in traditional Chinese and natural medicine. Our new finding that GUREE inhibits *h*MAO-B selectively is supported by our previous finding for its *h*MAO-B inhibition [17]. Interestingly, GUR was more selective than *Glycyrrhiza* glabra in our screen. Reported *Glycyrrhiza uralensis* different active constituents from other *Glycyrrhiza* genuses may influence its MAO-B selective inhibition [53]. GUR contains unique phytochemicals including isoprenylated phenolics [54] flavonoids, chalcones, and triterpene saponins [55]. Chalcone isoliquiritigenin, is an inhibitor for MAO-B [56] with multifunctional anti-inflammatory, antioxidant, cytoprotective [57] cellular detoxification system activator [58] and anti-apoptotic [59] anti-amyloid-β toxicity [60] neuroprotective properties. GUR total flavonoid extracts showed neurogenesis protective effect in depressed rats model [61]. The flavonoid liquiritin showed antioxidant and antiapoptotic neuroprotective effects in mice [62] and ameliorated depression in rat model [63]. Its benzopyran dehydroglyasperin-C also showed neuroprotection [64]. Xiaoyaosan, a traditional herb combination containing GUR for chronic depression, was effective in both animal models and clinical trials [65,66]. Other multifunctional properties of GUR constituents included reducing pro-inflammatory cytokines, nitric oxide, reactive oxygen species, lipid peroxidation [67], and mitochondrial impairment [68]. Interestingly, GUREE reports covered its chemopreventive [69] and anti-diabetic properties [70]. Therefore, specifically investigating GUREE as a selective MAO-BI could be beneficial.

## 5. CONCLUSION

Natural products are abundant of MAOIs with MAO-B selectivity and PCSEE, PABEE, GUREE, and FAREE are sources of yet to define MAO-B specific natural inhibitors. These plants contain high varieties of pharmacologically unique active phytochemicals such as coumarins, terpenes, flavonoids, and alkaloids. Therefore, the current findings may lead to the discovery of novel selective MAO-B inhibitors to benefit PD patients and beyond. Future research is required to elucidate and understand the pharmacological actions of these extracts and their phytochemicals which are responsible for the selectivity of *h*MAO-B inhibition and, consequently, finding safe therapeutic compounds for neurodegenerative diseases such as PD.

## Figures and Tables

**Table 1:** The top relative inhibitors against *h*MAO-B (RI_B_) with > 1.5-fold at 1 mg/mL plant ethanolic extract are 12 out of 132 extracted plants. Values reported are % of residual activity of *h*MAO-A and *h*MAO-B isozymes, with RI_B_= %*h*MAO-A/%*h*MAO-B

*Botanical name -* part used	*h*MAO-A *±* SEM (%)	*h*MAO-B *±* SEM (%)	*Rank*ed RI_B_ (fold)	P level
*Glycyrrhiza uralensis –* root	62.4±8.6	10.6±2.0	5.9	****
*Phellodendron amurense –* bark	18.2±1.2	3.5±0.01	5.3	**
*Camellia sinensis-* leaf	44.6±1.8	15.3±2.5	2.9	****
*Ferula assafoetida –* resin	37.3±5.2	14.0±0.1	2.7	****
*Piper nigrum –* fruit	41.4±2.3	18.0±0.3	2.3	****
*Baptisia tinctoria –* root	18.7±1.3	8.9±0.2	2.1	*
*Psoralea corylifolia –* seed	12.1±0.7	6.5±0.2	1.9	*
Phoenix dactyliferav- fruit	100.0±4.9	54.3±2.0	1.8	****
*Origanum majorana –* herb	35.4±0.2	19.6±1.7	1.8	**
*Magnolia denudate –* flower	35.3±1.1	19.9±0.1	1.8	**
*Lycopus lucidus* – rhizome	43.8±0.7	26.3±3.3	1.7	***
*Curcuma longa –* rhizome	44.0±0.8	28.3±1.4	1.6	**

Significance of difference between hMAO-A and hMAO-B% was determined using two-way ANOVA followed by Sidak’s multiple comparisons test.

* p ≤ 0.05,

** p < 0.01,

*** p < 0.001,

**** p < 0.0001

**Table 2:** The top relative inhibitors against *h*MAO-A (RI_A_) with > 1.5-fold at 1 mg/mL plant ethanolic extract are 11 out of 132 extracted plants. Values reported are % of residual activity of *h*MAO-A and *h*MAO-B isozymes, with RI_A_= %*h*MAO-B/%*h*MAO-A

*Botanical name -* part used	*h*MAO-A *±* SEM (%)	*h*MAO-B *±* EM (%)	*Rank*ed RI_A_ (fold)	P level
*Clematis trifoliate –* fruits	1.8±0.1	41.1±0.3	23.0	****
*Dryopteris crassirhizoma –* rhizome	5.5±0.1	38.1±0.8	6.9	****
*Tilia europaea* – leaf	18.5±1.8	36.6±0.5	2.0	****
*Zanthoxylum bungeanum* – seed	12.8±0.5	24.5±0.2	1.9	***
*Lindera aggregata* – root	17.1±1.5	32.0±0.2	1.9	****
*Laurus nobilis –* leaf	16.7±3.0	29.6±0.4	1.8	****
*Agrimonia pilosa* – herb	23.7±0.2	39.9±0.4	1.7	****
*Helichrysum foetidum –* flower	17.7±1.4	28.7±0.01	1.6	***
*Sargentodoxa cuneate* – stem	15.9±2.7	25.8±0.1	1.6	**
*Caesalpinia sappan –* bark	16.8±3.0	27.2±0.5	1.6	**
*Salvia apiana –* leaf	24.2±4.0	37.7±1.6	1.6	****

Significance of difference between hMAO-A and hMAO-B% was determined using two-way ANOVA followed by Sidak’s multiple comparisons test.

** p < 0.01,

*** p < 0.001,

**** p < 0.0001

## ABBREVIATIONS

EEs: Ethanolic extracts
RS_B_: Relative selectivity for hMAO-B inhibition
tyr. HCl: p-tyramine HCl benz
HCl: Benzylamine HCl
SNpc: Substantia nigra pars compacta
SN: Substantia nigra
RI: Relative inhibition
RI_B_: Relative hMAO-B inhibitor
AD: Alzheimer’s disease
DEP: Selegiline (Deprenyl^®^)
H_2_O_2_: Hydrogen peroxide
EOH: Ethanol
PCS: Psoralea corylifolia seeds
PAB: Phellodendron amurense barks
FAR: Ferula assafoetida resins
GUR: Glycyrrhiza uralensis roots
PCSEE: Psoralea corylifolia seeds ethanolic extract
PABEE: Phellodendron amurense barks ethanolic extract
FAREE: Ferula assafoetida resins ethanolic extract
GUREE: Glycyrrhiza uralensis roots ethanolic extract
MAO: Monoamine oxidase
hMAO-A: Recombinant human monoamine oxidase-A
hMAO-B: Recombinant human monoamine oxidase-B
HTS: High throughput screening
NE: Norepinephrine
